# Communicating projected survival with treatments for chronic kidney disease: patient comprehension and perspectives on visual aids

**DOI:** 10.1186/s12911-017-0536-z

**Published:** 2017-09-21

**Authors:** Frances Dowen, Karishma Sidhu, Elizabeth Broadbent, Helen Pilmore

**Affiliations:** 1 Department of Nephrology, Auckland City Hospital, 2 Park Road, Grafton, Auckland, 1023 New Zealand; 2 Department of Physiology, Auckland City Hospital, Auckland, New Zealand; 3 School of Medicine, University of Auckland, Auckland, New Zealand

**Keywords:** Visual aids, Survival, Communicating risk, Graphs

## Abstract

**Background:**

Mortality in end stage renal disease (ESRD) is higher than many malignancies. There is no data about the optimal way to present information about projected survival to patients with ESRD. In other areas, graphs have been shown to be more easily understood than narrative. We examined patient comprehension and perspectives on graphs in communicating projected survival in chronic kidney disease (CKD).

**Methods:**

One hundred seventy-seven patients with CKD were shown 4 different graphs presenting post transplantation survival data. Patients were asked to interpret a Kaplan Meier curve, pie chart, histogram and pictograph and answer a multi-choice question to determine understanding.

**Results:**

We measured interpretation, usefulness and preference for the graphs. Most patients correctly interpreted the graphs. There was asignificant difference in the percentage of correct answers when comparing different graph types (*p* = 0.0439). The pictograph was correctly interpreted by 81% of participants, the histogram by 79%, pie chart by 77% and Kaplan Meier by 69%. Correct interpretation of the histogram was associated with educational level (*p* = 0.008) and inversely associated with age > 65 (p = 0.008). Of those who interpreted all four graphs correctly, there was an association with employment (*p* = 0.001) and New Zealand European ethnicity (*p* = 0.002).

87% of patients found the graphs useful. The pie chart was the most preferred graph (p 0.002).

The readability of the graphs may have been improved with an alternative colour choice, especially in the setting of visual impairment.

**Conclusion:**

Visual aids, can be beneficial adjuncts to discussing survival in CKD.

## Background

The prevalence of end stage renal disease is increasing worldwide. Mortality in end stage renal disease (ESRD) can be higher than in many malignancies [1]. Treatment options for patients with ESRD include dialysis, transplantation and active supportive care. Median survival on dialysis, for those commencing treatment aged 45–64 is 6.6 years in Australia and 5.5 years in New Zealand. In the 65–74 age group, median survival is 4.3 years in Australia and 3.7 years in New Zealand [2]. Prevalent dialysis patients aged 65–69 in the US have an expected 4.65 year life expectancy [3].For suitable patients, transplantation has been shown to result in a significant survival benefit [4]. There is no data about the optimal way to present information about projected survival to patients with ESRD.

US data has shown that 97% patients, who may be suitable for renal replacement therapy, want to know about life expectancy [5] and a local survey found that 93% nephrologists in Australia and New Zealand felt a tool to show patients their projected survival in the setting of assessment for transplantation and pre-dialysis counselling would be useful in addition verbal explanation (Pilmore H, Personal Communication 2013). Increasing emphasis on patient centred decision making highlights the importance of effective communication of risks, benefits and complications of treatments. Disclosure of such information has been shown to generate trust, increase patient autonomy and possibly increase compliance [6].

Visual aids have been found to be useful adjuncts to numerical data in promoting understanding of risk of stroke and myocardial infarction in a study conducted on randomly selected households in the US and Germany [7]. Graphical representation of data has been shown to be more easily understood by patients than narrative alone in surgery and oncology [8]. Previous studies examining communication of risk estimates to a variety of participants, including university students, cancer patients and community volunteers over the age of 50, have shown a preference for histograms over Kaplan Meir curves and pictographs [9–11]. The development of patient decision aids identifies that visual formats are a key component of communicating risk estimates [12]. It has also been shown that in patients with limited non-native language proficiency, the use of visual aids reduced focus on the number of patients in the data set shown who had died, and increased awareness of overall cohort size in relation to mortality [13]. One study has developed visual aids specifically for use in communicating risk estimates in ESRD, however this is restricted to pictographs and does not investigate the success of different visual formats [14]. We aimed to determine patient comprehension of graphs showing survival information, in the setting of chronic kidney disease (CKD), and which visual method of explaining projected survival was preferable.

## Methods

### Participant recruitment and selection

Research ethics board approval was obtained prior to commencement of the project (NZ Health and Disability Ethics Committee 15/CEN/AM01). One hundred and seventy seven patients with CKD, on dialysis or post renal transplantation, were surveyed in the outpatient clinic setting, between August 2015 and February 2016. Clinics used for data collection were general nephrology, dialysis, transplant and transplant assessment clinics. All patients participating had CKD (eGFR based on Modification of Diet in Renal Disease equation) or were undergoing treatment with renal replacement therapy and sampling strategy was based on willingness to participate in the study. All patients attending routine clinic appointments or dialysis sessions during the study period were asked by their lead physician if they were willing to participate in a patient survey after their clinical appointment was completed. Those who were willing then received a verbal explanation of the project by a researcher, in addition to written information, and all gave informed consent. Patients who were under the age of 18, unable to read the graphs due to visual impairment or too unwell to participate were excluded. All non-English speaking patients are provided with an interpreter for clinic appointments and 6 patients (3%) completed the survey with the help of an interpreter. Patients were welcome to have any support people they wished in attendance whilst completing the survey.

### Data collection

**Fig. 1:**
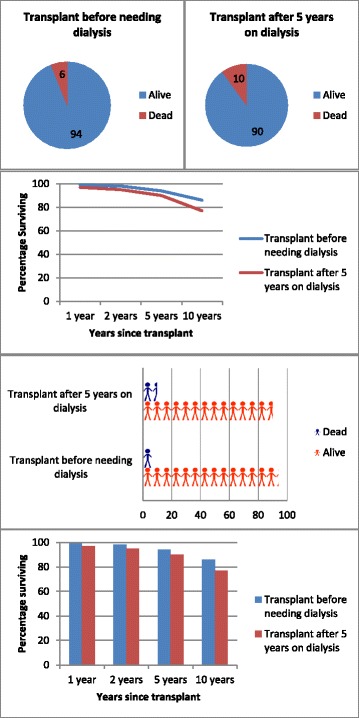
Example of the visual aids shown to patients. All four types of graph for one scenario are shown; pie chart, pictograph, histogram and Kaplan Meier curve. Scenario: Percentage of patients alive after transplantation, comparing transplantation after 5 years on dialysis, to transplantation before ever requiring dialysis

Participants were presented with four different clinical scenarios with accompanying graphs, showing risk, comparative treatment efficacy and treatment benefit. Each patient was asked to interpret a Kaplan Meier curve, a pie chart, a histogram and a pictograph (example scenario and graphs in Fig. 1) and answer a multi-choice question to determine their understanding of the graph. All scenarios had an option in the multi choice question stating ‘I don’t understand this graph’.The scenarios were: 1) Percentage of patients alive after a kidney transplantation, 2) Percentage of patients alive after kidney transplantation, comparing people who get a living donor transplant with people who get a deceased donor transplant, 3) Percentage of patients alive after transplantation, comparing smokers, ex- smokers and people who have never smoked, 4) Percentage of patients alive after transplantation, comparing transplantation after 5 years on dialysis, to transplantation before ever requiring dialysis. Participants were presented with 4 possible answers to the question ‘what does this graph show’ for each scenario, from which they were asked to choose the one that they thought was correct. As an example, the multi choice options given for the scenario shown in Fig. 1; ‘Percentage of patients alive after transplantation, comparing transplantation after 5 years on dialysis, to transplantation before ever requiring dialysis’ were a) That people who have a transplant before needing dialysis live longer, b) That people who have a transplant after 5 years on dialysis live longer, c) That it makes no difference to survival whether dialysis was needed before transplant, d) I don’t understand this graph. The pie charts show 5 year post transplantation data and this was stated in the survey. The survey design was formulated based on a number of publications examining the optimal way to present data to patients [8–11]. It was created with the assistance of staff in the Dept. of Psychological Medicine, University of Auckland. There was equal graph distribution per scenario so each graph type was assessed equally.

All of the scenarios contained generic post transplantation survival data with survival projections, (based on Australia and New Zealand Dialysis and Transplant Registry (ANZDATA) post transplantation survival data [2]) that were not specific to the individual patient. Each of the four scenarios had a graph of each type created to accompany it, and the graph type was randomly assigned to the scenario, so that each patient received one of each type of graph. The graphs were all shown in blue and red colour combinations. The smoking status scenario had 3 data elements (smoker, non-smoker and ex-smoker) so this graph also contained a green data element, in addition to blue and red. Information was presented to patients in paper form, with one scenario and graph and per page. A member of the research team was present throughout the survey completion and offered an explanation of each graph if requested, however no further assistance was offered to aid interpretation. If an ambiguous answer was given, the participants were offered an explanation, if they had not already received one, and if their answer remained ambiguous it was marked as incorrect.

We collected demographic data including educational level and employment status. Following completion of the scenarios, patients were asked whether they found the graphs helpful in understanding the information and any preference held, they were also encouraged to give free comments which were recorded. All patients were offered the opportunity to discuss the issues raised by the survey with their physician.

### Data analysis

#### Power calculation and statistical analysis

In order to detect a 5% difference with 90% confidence in patient preference for the four different graphical types, and the percentage of correctly interpreted graphs, a sample size of 177 patients was required. Baseline data are reported as a median with interquartile range or a percentage of the population examined (%).A Chi-squared test was used to determine which type of graph was stated as preferred by patients. The Cochran’s Q test was used to compare the percentage of correct answers to each scenario for each type of graph. Non parametric testing using a Kruskal-Wallis one-way analysis of variance was used to determine which factors were associated with a correct answer for each graphical type and for those participants who achieved all four correct answers. Statistical analyses were performed using the statistical package SAS version 9.3 (SAS Institute, Cary, NC). Power calculation was done with G-Power [http://www.gpower.hhu.de/en.html]. A *p*-value of <0.05 was considered significant.

#### Qualitative analysis

After completion of the survey all participants were asked if they had found the graphs useful, any preference for a particular type of graph, and also asked for any free comments or feedback they had on the graphs. These were recorded in order to generate qualitative data. Relevant themes were identified amongst the free comments offered by participants through discussion and agreement amongst the co-investigators, alongside external advice from our acknowledged contributor for qualitative analysis. These themes were then analysed through further discussion to explore how they inter-related and impacted on our results. We were able to classify the comments into patient factors; how disease defined reaction to the images, image factors; the accessibility of the graphs, and finally the importance of preserving hope and how the images shown impacted on this.

## Results

### Participant characteristics

All participants reviewed all 4 scenarios. Of the 177 participants, the median age was 55 years, comprising of 56% male and 44% female. The largest ethnic group represented were New Zealand European. Less than half of patients were employed and most had achieved at least a secondary level of education. Forty seven participants (27%) had previously undergone renal transplantation, which was functioning at the time of the study, and 38 (21%) were having dialysis treatment. Demographic data can be seen in Table 1.

**Table 1:** Demographic data of participants

	Number of patients
Age	
< 20	0
20–35	19 (11%)
36–50	48 (27%)
51–65	59 (33%)
> 65	51 (29%)
Gender	
Male	100 (56%)
Female	77 (44%)
Ethnicity	
NZ European	82 (46%)
Pacific	39 (22%)
Maori	23 (13%)
Asian	20 (11%)
Other	13 (7%)
Employed	75 (42%)
Educational level	
Primary	7 (4%)
High School/College	99 (56%)
Tertiary	70 (40%)
Nil	1 (0.5%)
eGFR^a^	
> =90	9 (7%)
60–89	17 (12%)
45–59	18 (13%)
30–44	23 (17%)
15–29	43 (31%)
< 15	28 (20%)
Cause of CKD	
Diabetes	59 (33%)
Glomerulonephritis	50 (28%)
Hypertension	14 (8%)
Other	36 (20%)
Unknown	18 (10%)
Dialysis	38 (21%)
Previous transplant^b^	47 (27%)
Diabetes	70 (40%)
Total	177

^a^In non-dialysis patients only, data unavailable for 1 patient

^b^Information unavailable for 1 patient

### Comprehension

**Fig. 2:**
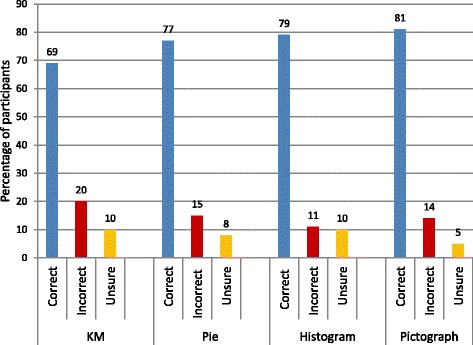
Interpretation of graphs

Most patients were able to interpret the graphs correctly (Pictograph = 81%, Histogram = 79%, Pie Chart = 77%, Kaplan Meier = 69%; Fig. 2) with a significant difference in the number of patients interpreting each graph correctly (*p* = 0.0439).

The ability to correctly assess graphs was not associated with gender, ethnicity, or exposure to dialysis or kidney transplantation (Table 2). Those aged >65 were less likely to correctly interpret the histogram (*p* = 0.008) than those under the age of 65. Patients who had attended high school or a tertiary institution appeared to be more likely to correctly interpret graphs with significantly more correct than incorrect answers for the use of histograms (p = 0.008).

**Table 2:** Percentage of correct answers for each graph comparing demographic groups

Correct Answers				
	Pictograph	Histogram	Pie Chart	Kaplan Meier
Overall	143 (81%)	140 (79%)	136 (77%)	122 (69%)
Age > 65	42 (81%)	36 (69%)	38 (73%)	33 (63%)
*p value*	*p 0.5*	*p 0.008*	*p 0.05*	*p 0.5*
Gender				
Male	83 (83%)	84 (84%)	77 (77%)	74 (74%)
Female	60 (78%)	56 (73%)	59 (77%)	49 (63%)
*p value*	*p 0.2*	*p 0.4*	*p 0.2*	*p 0.5*
Educational Level				
Primary/Nil	6 (75%)	4 (50%)	2 (25%)	4 (50%)
High School/College	79 (80%)	77 (78%)	75 (76%)	64 (65%)
Tertiary	59 (84%)	59 (84%)	59 (84%)	55 (79%)
*p value*	*p 0.09*	*p 0.008*	*p 0.08*	*p 0.9*
Ethnicity				
NZ European	69 (84%)	65 (79%)	70 (85%)	69 (84%)
Pacific	31 (79%)	30 (77%)	23 (59%)	22 (56%)
Maori	16 (70%)	19 (83%)	16 (70%)	11 (48%)
Asian	18 (90%)	18 (90%)	16 (80%)	12 (60%)
Other	9 (69%)	8 (62%)	11(85%)	9 (69%)
*p value*	*p 0.4*	*p 0.7*	*p 0.4*	*p 0.7*
Employment	63 (84%)	66 (88%)	62 (82%)	61 (81%)
*p value*	*p 0.7*	*p 0.4*	*p 0.6*	*p 0.2*
Cause of CKD				
Diabetes	44 (75%)	46 (78%)	38 (64%)	35 (59%)
Glomerulonephritis	42 (84%)	39 (78%)	40 (80%)	40 (80%)
Hypertension	12 (86%)	11 (79%)	11 (79%)	9 (64%)
Other	30 (83%)	31 (86%)	34 (94%)	28 (78%)
Unknown	15 (83%)	13 (72%)	13 (72%)	11 (61%)
*p value*	*p 0.7*	*p 0.3*	*p 0.4*	*p 0.9*
Diabetes	52 (74%)	55 (79%)	50 (71%)	36 (51%)
*p value*	*p 0.2*	*p 0.8*	*p 0.2*	*p 0.8*
On dialysis	27 (71%)	29 (76%)	30 (79%)	24 (63%)
*p value*	*p 0.1*	*p 0.8*	*p 0.2*	*p 0.5*
Previous transplant	41 (87%)	40 (85%)	37 (79%)	37 (79%)
*p value*	*p 0.9*	*p 0.8*	*p 0.9*	*p 0.8*

Hypothesis tested being that demographic variables would be associated with ability to correctly interpret different types of graph

The New Zealand European group contained the greatest proportion of employed participants (Table 3). No participants with a primary level of education were employed, whereas 39% with secondary education and 61% with tertiary education were employed.

**Table 3:** Educational level and Employment status by Ethnicity

Ethnicity	Primary Education	Secondary Education	Tertiary Education	Employed	Total
NZ European	3 (4%)	43 (52%)	36 (44%)	43 (52%)	82
Pacific	3 (8%)	25 (64%)	11 (28%)	9 (23%)	39
Maori^a^	1 (4%)	16 (70%)	5 (22%)	7 (30%)	23
Asian	0	10 (50%)	10 (50%)	10 (50%)	20
Other	0	5(38%)	8 (62%)	6 (46%)	13

^a^One patient received no formal education

Of the 83 participants who interpreted all four graphs correctly (47%), no significant link was found to age > 65, gender, educational level, cause of CKD, dialysis treatment or previous transplant (Table 4). There was however a statistically significant association with ethnicity, employment and diabetes. New Zealand European participants were more likely to interpret all the graphs correctly (*p* = 0.002), as were those in employment (*p* = 0.001). People with a diagnosis of diabetes were less likely to interpret all four graphs correctly (*p* = 0.003).

**Table 4:** All four correct answers by demographic variable

Variable	number in group with all correct	*p* Value
Age > 65	20 (24%)	*0.2*
Gender		
Male	50 (60%)	*0.2*
Female	33 (40%)	
Ethnicity		
NZ European	52 (63%)	
Pacific	11 (13%)	*0.002*
Maori	6 (7%)	
Asian	9 (11%)	
Other	5 (6%)	
Educational Level		
Primary	2 (2%)	
High School/College	40 (48%)	*0.2*
Tertiary	41 (49%)	
Employment	46 (55%)	*0.001*
Cause CKD		
Diabetes	22 (27%)	
Glomerulonephritis	27 (33%)	*0.2*
Hypertension	6 (7%)	
Other	21 (25%)	
Unknown	7 (8%)	
Diabetes	28 (34%)	*0.003*
Dialysis	15 (18%)	*0.2*
Previous Transplant	29 (35%)	*0.06*

Hypothesis tested being that patient demographics would be associated with likelihood of interpreting all four graphs correctly

### Usefulness

Eighty seven percent of patients found the graphs useful in assisting their understanding of the survival data. Eight percent did not find the graphs particularly helpful and 5% stated no preference for information delivery between visual and narrative.

### Patient preference

The pie chart was stated to be the most popular graph of those that had a preference (36%) and the Kaplan Meier the least preferred (12%), *p* = 0.001. Twenty two percent of stated preferences favoured the pictograph but 15% specifically commented that this graph was the least easy to read.

### Qualitative data

**Fig. 3:**
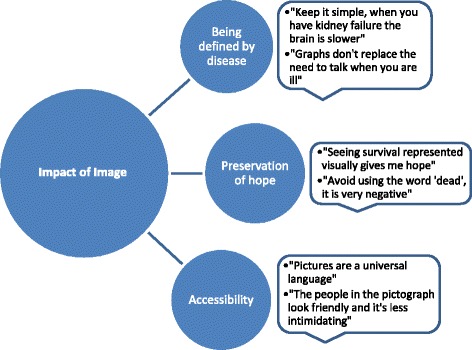
Thematic analysis

Eighty three participants (47%) recorded comments about the data. We identified three main themes: being defined by chronic disease, preservation of hope, and accessibility of data (Fig. 3). The majority of free comments (54%) concerned the accessibility of data, for example “pictures are a universal language”. Eleven percent specifically related to the preservation of hope, for example “Avoid the use of the word ‘dead’, it is very negative”. Finally, 7% of comments reflected the fact that interpretation of the graphs was affected by underlying disease, for example, “Keep it simple, when you have kidney failure the brain is slower, so you need simple, clear graphs”.

## Discussion

This is the first study examining the use of graphs to portray survival outcomes after kidney transplantation to patients with renal failure. We have demonstrated that 87% patients found a graphical representation of survival data a useful tool in augmenting their understanding. Most patients were able to correctly interpret the graphs, although the Kaplan Meier was the most likely to be incorrectly interpreted, while over 80% were able to correctly interpret the pictograph.

The interpretation of graphs appeared to be more likely to be correct in those with at least a high school level of education. It is possible that this is due to better graphical literacy with higher levels of education. Interestingly the pictograph appeared more easily understood in those with only primary education although this did not reach statistical significance. Correct interpretation of the histogram was associated with a higher educational level and inversely associated with age > 65, possibly reflecting greater familiarity with this type of chart amongst younger and more educated participants.

Correct interpretation of all four graphs was significantly linked to employment and being New Zealand European. This may be on the basis of cultural and social backgrounds and relate to increased exposure to graphical images. New Zealand Europeans were more likely to be employed. Employment was more likely with higher levels of education, which may explain this data. Diabetics were less likely to interpret all four graphs correctly, which may relate to associated visual problems and general health and wellbeing in the setting of chronic multisystem disease with microvascular complications.

There was a clear preference for pie charts with 36% of comments favouring this graph type. The Kaplan Meier was the least preferred and, though 22% preferred the pictograph, this was also the most disliked graph. Previous studies have shown patient preference for histograms, compared to Kaplan Meier curves and pictographs, in an all-female population [9]. Vertical bars have been shown to be the fastest and most accurately interpreted visual aid, in a population aged over 50, when comparing with horizontal bars, pie charts, digits, systematic and random ovals [11]. One study of surgical patients aged 50–90 suggests that Kaplan Meier curves were the most preferred option, with pictographs being the least preferred and no significant difference was noted related to sex or educational background [15]. Our results corroborate that pictographs are poorly favoured in communicating information to patients. The preference for pie charts is a new finding.

Comments made by patients were illuminating and helped us to appreciate the impact of image on understanding, memory and the language barrier. Thematic analysis led us to identify that many patients feel defined by their disease, commenting on the need for simple images and the desire for physician narrative alongside use of visual aids. Preservation of hope was highlighted as important, and some patients found that the visual representation of survival encapsulated this. The use of the word ‘dead’ was portrayed as detrimental within this theme. Visual data seemed more able to breach the language barrier and provide accessibility for a wide spectrum of patients. Within the accessibility theme, the domains of colour and framing were prominent. Colour was highlighted as an important factor in the physical and emotional response to the image. Red was perceived as a ‘warning colour’ and the choice of colours needs to be appropriate to avoid ambiguity in colour blindness (avoidance of red/green colour combinations). Several diabetic patients stressed the importance of clear blocks of colour in those with diabetic eye disease. It has previously been shown that the use of blue/white and blue/yellow colour combinations did not alter interpretation of an image [11]. Advice from the Blind Foundation regarding the use of visual aids in those with low vision is to increase contrast and reduce glare [16]. A blue/yellow colour combination, with colour contrast validated according to international standards, as recommended by Blind Foundation, may be the most inclusive colour combination for visual aids in this setting. We were made aware of how the presentation of information may frame the data negatively or positively and thus alter the response and interpretation of an image, for example a half figure in the pictograph can be interpreted as a negative outcome as the person is incomplete. It is important that we are aware of framing so that we present data objectively. In future it may be beneficial to create pictograph units as whole symbols.

There were potential limitations to the study. The quality of the graphs may be improved with an alternative colour choice, as advised by the Blind Foundation. The participants had varying levels of knowledge about the subject matter included in the survey. This was due to their varying stages of kidney disease, stage in the process of renal replacement therapy education, and engagement with the health care system. Some patients may have been able to answer some of the questions from prior knowledge, rather than relying on the graphs. This was especially true of the smoking scenario which some answered intuitively. The semantics of the multi choice answers were a limitation and it may have been more suitable to state, for example (using the scenario in the graphs in Fig. 1), ‘that *more* people who have a transplant before requiring dialysis live longer’. The answers were simplified to help make the survey more accessible to the general public, but we acknowledge that this may have made the multi choice answers too absolute. There was a strong emotional response to the graphs from some participants. By using non-medical scenarios we could have avoided the emotive element, however this would have been a less relevant and potentially less engaging process. Although the surveys were carried out by one researcher and the explanation of graphs was similar for each patient, there was no formal standardised explanation for each graph. In order to help standardise this, and gain further information into the accessibility of the graphs, it may be useful to collect data on the number of explanations required. In reality, a physician-patient relationship would demand that the explanation be tailored to the needs of the individual. As this study moves forward we would value discussing an explanation strategy with the physicians using the visual aids and audit the employment and success of this.

## Conclusion

We believe our study will encourage use of visual data in supporting patient understanding and choice in ESRD. While the pictograph was the most correctly interpreted, the use of pie charts appears to be the most popular in this patient cohort. We plan to begin using visual aids, with a preference for pictographs and pie charts, within our department as part of counselling for treatments for ESRD. The results of this study will inform this process and the success of these visual aids will be audited. The findings from this study have the potential to shape our interactions, empower our patients, and further develop patient centred and directed care.

## Abbreviations

ANZDATA: Australia and New Zealand dialysis and transplant registry
CKD: Chronic kidney disease
ESRD: End stage renal disease
